# Integration of Information Technologies in Clinical Studies in Nicaragua

**DOI:** 10.1371/journal.pmed.0040291

**Published:** 2007-10-23

**Authors:** William Avilés, Oscar Ortega, Guillermina Kuan, Josefina Coloma, Eva Harris

## Abstract

The authors report their experience of integrating information technologies in clinical and epidemiological studies of dengue infection in Nicaragua.

Over the past several years, information technologies have been increasingly used in health applications in developing countries [1–5]. Here we report our experience integrating a number of information and communication technologies (ICTs) into clinical and epidemiological studies in Nicaragua. Since 2004, our team (from the Nicaraguan Ministry of Health, the Sustainable Sciences Institute, and the University of California, Berkeley) has established a pediatric dengue cohort study (PDCS) in Managua, Nicaragua. Currently in its third year, the PDCS follows 3,800 children aged two to twelve with the aim of characterizing the natural history of dengue transmission, obtaining biological samples for vaccine safety research, and establishing appropriate infrastructure for future dengue vaccine trials.

The PDCS operations are based in a Health Center where cohort children receive all primary care and are screened for dengue. Cohort children are visited in their homes for collection of annual blood samples, convalescent follow-up of suspected dengue cases, and periodic surveys. All samples are processed, catalogued, and stored at the National Virology Reference Laboratory, where serological, virological, and molecular diagnosis of dengue is performed. Finally, the Sustainable Sciences Institute, a nonprofit organization, is in charge of the project administration, coordination, data analysis, and quality control (QC) informatics and assurance.

Managua, the site of the study, represented both a great challenge and an opportunity. As in many other cities in the developing world, frequent interruptions in electrical, phone, and Internet service, high temperatures and humidity, and the absence of street names and house addresses were obvious obstacles to be overcome. Additional hurdles included a time-consuming and ineffective manual process for locating patient medical records at the Health Center, the absence of a formal QC plan for clinical information and biological samples, and a rudimentary organizational system in the laboratories.

To overcome these challenges, we implemented a series of low-cost yet cutting-edge ICTs. The systems were initially developed to facilitate the annual sample collection and home visits, but have now been applied in an integrated manner to other aspects of the study as well as to numerous additional routine procedures in the Health Center and Virology Laboratory (Table 1). They have not only greatly aided fieldwork but have improved QC at all levels far beyond our initial objectives and expectations. Using readily available standardized hardware and software, our Nicaraguan informatics engineer programmed, designed, and supervised the implementation of a customized ICT system that includes geographic information systems (GIS) to map and easily locate study participants' homes, personal data assistants (PDAs) for paperless data entry and wireless data upload, barcode printing and scanning for tracking participant information and specimens, fingerprint scanning for facilitating patient identification and follow-up, low-cost communication systems (Skype, two-way radios), scanners for electronic backup of all documents, and computerized information systems with integrated databases for information management and control (Figure 1).

**Table 1:**
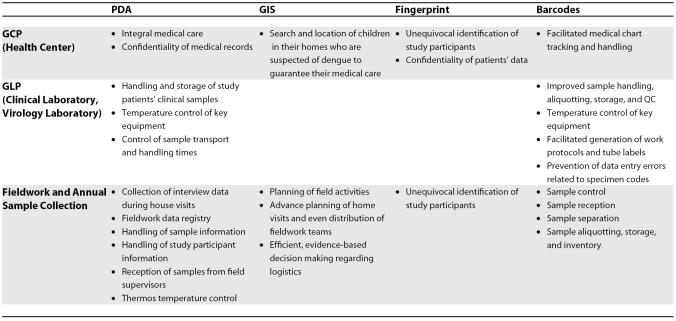
Advantages of ICTs to Facilitate GCP, GLP, and Fieldwork

**Figure 1:**
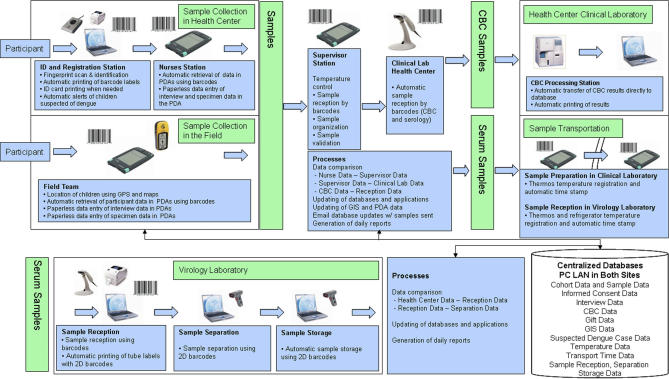
Integration of Information Technologies in Fieldwork, the Health Center, and the Virology Laboratory Patient identification and sample tracking from time of collection though storage in the Virology Laboratory. CBC, complete blood cell count; PC LAN, personal computer local area network.

During two and a half years of operation, we found that the use of these technologies greatly streamlines information flow and accessibility, improves the quality of data and QC procedures, and reduces operational costs. As a result, we have witnessed the tremendous potential for using ICTs to bolster the public health infrastructure in resource-limited developing country settings. The integration of these ICTs for different applications (fieldwork, good clinical practice [GCP], good laboratory practice [GLP], quality control, and communication) is described below.

## Fieldwork

The geographic localization of the vast majority (more than 95%) of cohort participants has been mapped using GIS—very useful in a city with unplanned urbanization and lack of addresses and street names. Hand-held global positioning system (GPS) devices are used to readily locate participants' houses during routine home visits and collection of convalescent sera from suspected dengue cases. GIS has also been instrumental in the daily logistics of planning fieldwork and for informed decision making regarding efficient deployment of nurse teams throughout the study territory. For example, frequent progress reports of advances in house-to-house visits throughout the day greatly facilitates planning the next day's targeted visits and recapture strategy. Detailed geographic knowledge allows a more efficient selection of homes to be visited, despite the lack of street addresses.

A yearly blood sample is drawn from all 3,800 study participants. While in the first year of the study, medical data were recorded manually on paper, by the second year, the use of PDAs allowed for direct data entry during house visits. Data collected by field teams are immediately beamed to a supervisor's PDA, returned to the Health Center, and transferred to the main database, permitting real-time assessment of daily progress and evaluation for the next day's planning. Instead of manually entering the code of each specimen tube, samples are now tracked by barcode throughout their journey from point of collection through the clinical laboratory in the Health Center to the Virology Laboratory; this also allows control of temperature and time of transport. Barcode scanning of identification cards is also used to identify the children. After implementation of the integrated ICT system (Figure 1), study personnel dedicated to fieldwork during the annual sample collection process (including logistical support, data entry, and nursing staff) decreased from 23 to 12, while data entry personnel were reduced from five to one. Importantly, improved supply and inventory control, the use of two-way radios rather than cell phones, the implementation of PDA-based paperless questionnaires, and reduced personnel costs have resulted in substantial savings.

## Good Clinical Practice

The use of low-cost ICTs has allowed the study to maintain compliance with international GCP-ICH (International Conference for Harmonization) standards for recording, handling, and storing study information and assuring patient confidentiality (Table 1). We have implemented digital fingerprint scanners and photo- and barcode-based patient identification cards to identify study participants and retrieve medical records at the Health Center's admissions desk, dramatically reducing the time required for these processes (W. Avilés et al., data submitted for publication). Barcodes have also facilitated efficient medical chart processing and patient flow. Before a medical chart exits or enters a Health Center location, it is scanned and the location to which it will be moved and the person responsible for it are recorded in the database, improving the ability to locate patients' charts at any given time and ensuring that records have completed the data entry process properly. Patient flow has been improved by speeding up the process of patient identification, medical chart localization, and movement between areas of medical attention. In addition, physicians now use PDAs to record medical histories of study patients and automatically upload the information into the network database. This facilitates data entry by avoiding the use of paper forms that need to be entered in duplicate, and permits real-time data quality control and analysis.

Information technology applications also enable researchers to uphold patient confidentiality standards. Barcodes and fingerprint scanners permit study personnel to retrieve participant codes to confirm patient identity and locate clinical information without using patient names. To protect participants' identities, a comprehensive password system is used that includes the following attributes: (1) for entry into study databases, networks, servers, and PDAs, study personnel must enter their unique passwords, which only allow access to the privileged information relevant to their position; (2) to increase security, database passwords are changed at least once every six months. This system represents a considerable improvement in safekeeping and confidentiality of patient information in all sites, compared to paper records.

## Good Laboratory Practice

**Figure 2:**
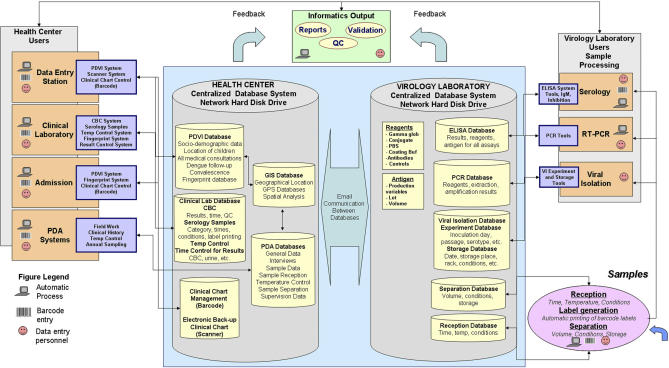
Design and Implementation of Information Technology Systems in the Health Center and Virology Laboratory and Interrelation between Databases CBC, complete blood cell count; IgM, immunoglobulin M; PBS, phosphate-buffered saline; PDVI, Pediatric Dengue Vaccine Initiative; VI, virus isolation.

According to international standards, the purpose of GLP is “…to provide a managerial tool to ensure a sound approach to the management, including conduct, reporting and archiving, of laboratory studies” [6]. The use of low-cost ICTs has ensured that the PDCS maintains GLP from sample collection to reception, separation, processing, and storage (Table 1). The system was designed in such a way as to enable reagent lot control, generation of electronic work protocols, assay validation, and confirmation of final results (Figure 2; see Text S1 for more details).

At the Health Center's clinical laboratory, blood samples are obtained from children suspected of infection with dengue virus. A barcode is created that identifies the study code, type of sample, and date. A complete blood count is performed on-site and results are transferred directly to the database computer, reducing potential data entry errors. Samples collected in the field or the Health Center are barcoded and immediately transported in a 4 °C thermos to the Virology Laboratory, where they undergo serological, virological, and molecular diagnosis. During transit, the temperature of the thermos and transport time are recorded in a PDA and registered in the database to monitor the condition of specimens during transit.

In the Virology Laboratory, the sample's information is automatically retrieved via the barcode and stored in the Sample Reception and Separation Databases, which print 2D barcode labels for all the aliquots generated and select the rack and freezer in which the sample will be stored. The location and volume information of each sample aliquot, which is updated as the supply is used, is now easily retrievable from the database. The system has been designed to allow instant access to information about samples (e.g., volume and number of working and archival aliquots, storage conditions, number of assays run, assay conditions including reagent lots, and work protocols). It also monitors whether control values and duplicate samples are within the acceptable range and records results, thus facilitating review, QC checks, and report generation. In addition, the temperatures of all freezers, refrigerators, and incubators are recorded via PDA twice daily, and graphical representations of temperature variation are generated.

A great improvement in the laboratory has been the implementation of organizational procedures to improve QC, minimize human error, and streamline protocols. A central server stores all databases, allowing three methods of information input: barcode, data entry, and automated entry (e.g., directly from a PDA, ELISA reader, etc.) (Figure 2). For instance, for the immunoglobulin M ELISA, barcodes on the sample tube are scanned to create the work protocol; cutoff values are automatically calculated from the controls, and based on this number, samples are automatically assigned a positive or negative outcome. These results are then examined and confirmed one-by-one by the technician and the Laboratory Director and uploaded to the central database, along with critical information such as antigen and control lots used in the assay. All study-related protocols (ELISAs, viral isolation, reverse transcriptase PCR) have been similarly automated. Since all the techniques are used to analyze samples from the PDCS as well as specimens from routine dengue surveillance and other dengue research studies, the code of the samples serves as the identifier that determines to which database the assay and result will be sent, allowing separate reports to be easily produced.

## Quality Control and Integrated Analysis

ICTs have also proven useful in the maintenance of QC standards at the level of immediate contact with the document or specimen, flow of documents and specimens between areas and sites, and database review. Automatic check functions and response confirmation are built into the PDA data entry programs; manual data are double-entered into databases containing check functions; and biweekly comparisons are performed between duplicate databases using comparison report software. QC queries are run daily and weekly on all databases to verify the quality of the data and correct any errors in real time. Full database reviews occur at least once every three months. Barcode-based applications for specimen processing allow daily confirmation that each sample has passed though all work stations properly. In addition, barcodes on documents allow verification that all procedures associated with those documents (e.g., letters of consent, medical charts, and forms) are properly executed.

Lastly, information technologies allow implementation of a system of work ownership. All personnel are assigned a code for database entry, supervision, and analysis. Using these codes, logs are maintained to control data management and information flow. Together, these methods ensure that data are promptly, accurately, and legibly recorded, frequently verified, and easily accessible for QC, integrated analysis, and reports.

## Communication

Poor landline phone service prompted the adoption of mobile phones for internal communications between study personnel. Although effective, cell phones are expensive in Nicaragua for both domestic and international communication. Therefore, we switched to free-of-charge Voice-over-Internet-Protocol technology (e.g., Skype) for international communications, including videoconferencing and instant messaging. Skype is now also used for local inter- and intra-site communication, and instant messaging has proven to be an efficient way to maintain real-time connectivity. Two-way radios, first implemented for field operations, are currently used daily within the Health Center for rapid communication. This improved connectivity has been a key component for QC and program management and has kept costs at minimum.

## Discussion

To best illustrate the advantages of integrating ICTs into developing country health applications, we chose the specific example of the PDCS; however, the systems described can clearly be implemented in any number of clinical studies and trials. Specific limitations of each technology and solutions found to overcome them are listed in Table 2. Overall, the limitations of ICTs in Nicaragua derive from the fact that much of the equipment is not available locally; however, most of the hardware is available through collaborators in industrialized countries or can be ordered directly via the Internet. Surprisingly, these technologies do not represent excessive costs (all devices range from ~US$100–US$500; for detailed costs see Table S1); rather, they are an investment with immediate return. One important aspect is the need for local informatics experts for development and custom integration of databases, continued support, and adaptation to new applications. Specifically, a programmer with experience in database and systems design, implementation, and maintenance is recommended, and this resident expertise is available in most if not all countries. Lastly, the logistics of operating our technology-based system in a setting where power outages are common is a challenge that we have dealt with through the use of rechargeable batteries and backup power generators.

**Table 2:**
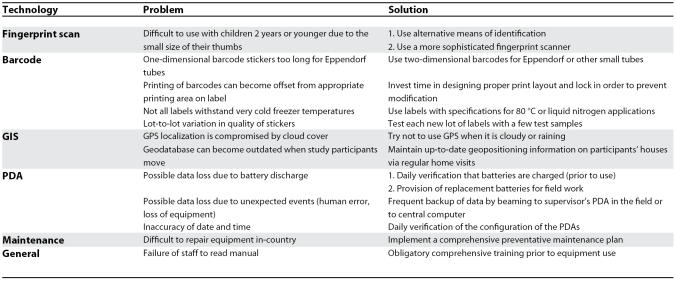
Limitations of ICTs and Solutions

We have demonstrated that the combined use of ICTs in clinical studies has numerous advantages, such as improved QC, streamlined laboratory and fieldwork procedures, increased efficiency and reduced costs, real-time analysis of data, local capacity building, and better quality of patient care. Importantly, the design and implementation of this system was driven by user needs in a highly resource-limited setting (Nicaragua is the second poorest country in the Western hemisphere). Local personnel have not only acquired the skills to master the new technology-based systems but can also train their peers in the use of ICTs. In many instances, this experience has awakened interest on the part of doctors, nurses, and technicians in accessing information via the Internet and participating directly in health research, thus providing the opportunity to grow professionally and to take leadership in solving their community's health problems. Importantly, the advances brought about by integration of ICTs should continue to be felt by the local population at the conclusion of the study, as these technologies and approaches are already being implemented in other areas of the Nicaraguan Ministry of Health, such as improving vaccination efficiency in the national immunization program and bettering prenatal care. In summary, we believe this project serves as a pioneer locally and perhaps globally with respect to the effective integration of ICTs in the health arena and the application of ICTs to achieve GCP, GLP, and QC compliance for clinical studies and trials.

## Supporting Information

Table S1 Detailed information about hardware and software, including prices. (96 KB DOC).

Text S1 Design and maintenance of the databases in the system. (53 KB DOC).

## Abbreviations

GCP: good clinical practice
GIS: geographic information system
GLP: good laboratory practice
GPS: global positioning system
ICT: information and communication technology
PDA: personal data assistant
PDCS: pediatric dengue cohort study
QC: quality control
