# Supplementary material for: Integration of Information Technologies in Clinical Studies in Nicaragua
Source: PLoS Med. 2007 Oct 23;4(10):e291. doi: 10.1371/journal.pmed.0040291 (PMC2039765; doi:10.1371/journal.pmed.0040291)
Supplement: Supplementary file 1 [file pmed.0040291.sd001.doc]

**Text S1. Design and maintenance of the databases in the system**

**I. Database design and maintenance**

**A. Database Design**

All the databases were created in Microsoft Access and are password-protected. They are relational databases separated according to their function and location in the server. The server validates users via passwords that define each user’s access privileges to the data. The common factor in all the databases is the ID code of each study participant, with which all the tables can be related, independent of the database of origin. When information is analyzed for reports or quality control, a database is used with the necessary tables linked to the original databases that contain the information, such that all the tables are accessible no matter where the original databases are, as long as the new database is linked to them.

**B. Database back-up**

According to established procedures, data entry personnel are in change of backing up all the databases of the study. This is done on the server by date as well as on external storage (CD or DVD) at least once a week.

**C. Error control**

To minimize errors, double data entry is performed. All data are entered twice, in separate databases. These databases are then compared to search for discrepancies; when one is found, it is corrected, and the process is repeated until the two databases are identical. This comparison is done using the EpiInfo “Check” program, and all the documentation generated by the program in html is saved (e.g., discrepancies encountered, dates, statistics of the comparison).

**II. Databases in the Health Center**

**A. Data Entry Area**

**General PDVI Database PDVI (General Cohort Data)**

Tables:

Sociodemographic data

Medical consults

Dengue follow-up

Convalecent data

Documentation of withdrawals

Data about home visits

Fingerprint data

Catalogs of linked data

This database has an interface developed in Visual Basic 6 (Microsoft) that permits validation of which user is using the databases, since each user has a unique password that defines his/her access privileges to the database. This allows registration of which user created the registry and the last user to modify it, along with the date of modification. Modifications to the database are also noted in a registry book, which includes who made the modification, who authorized it, dates, and signatures of all involved.

**Electronic back-up of medical charts**

The medical charts of the cohort children are scanned so as to maintain an electronic back-up of the original documents. When there are any modification, the medical chart is scanned again.

**Database of handling of medical charts**

Tables:

Medical charts

Movement of medical charts

Tables of linked data (personnel, location, etc.)

This system has forms in Access that allow registration of the movement of a medical chart within the Data Entry Area via barcode. It also allows verification of the medical chart of suspected dengue cases using “flags”. The location of a medical chart can be identified immediately, and this system also ensures that medical charts that come out of the medical consult are entered into the database. This database serves for comparison with the analogous database in the Admissions Office.

**B. Clinical Laboratory**

**CBC (complete blood count) database**

Tables:

CBC results

Laboratory personnel

Tables linked with general data for printing reports to be delivered to study participants’ parents

In the CBC database, the machine that performs CBC is connected to a PC that reads the results directly from the serial port and stores them in the database. This system was developed in Microsoft Visual Basic 6.0 and validates system users. It does not permit modification of data, only invalidation of the CBC result if something went wrong. The system notes when and by whom a CBC result is invalidated. It also allows review of all the CBC counts performed for each study participant.

**Serology database**

Tables:

Samples for serology

Laboratory personnel

This system is in Access and validates entry via username and password. Data is entered in duplicate (as explained above) to minimize errors. This database stores information about the samples sent to the Virology Laboratory and automatically prints the specimens’ barcode label that contains the code number, type of sample, category, and date.

**ID database**

Tables:

Linked general data

Linked digital fingerprint scans

This database serves for identification of the study participant using the fingerprint scan.

**Other databases in the Clinical Laboratory**

**Temperature database:** Registers the average temperature measured in the refrigerator in the Clinical Laboratory twice daily.

**Test results database:** Registers the tests performed in the Clinical Laboratory and the time taken to return the results to the patient.

**C. Admissions Office**

**Database of handling of medical charts**

Tables:

Medical charts

Movement of medical charts

Tables of linked data (personnel, location, etc.)

This system has forms in Access that allow registration of the movement of a medical chart within the Admissions Office via barcode. It also allows verification of the medical chart of suspected dengue cases using “flags”. The location of a medical chart can be identified immediately, and this system also ensures that medical charts that come out of the medical consult and are entered into the database are returned to the Admissions Office. This database serves for comparison with the analogous database in the Data Entry Area.

**ID database**

Tables:

Linked general data

Linked digital fingerprint scans

This database serves for identification of the study participant using the fingerprint scan.

**D. PDA database**

The systems that are used with PDAs were developed using Pendragon Forms 5.0 (and its own programming language) based in Microsoft Access. Palm Desktop is used to synchronize the devices with the computer when Palm devices are employed. More technical information can be found at: <http://www.pendragon-software.com/>. Almost all the forms used in the PDAs are for download into the computer. Forms that serve to review information are set as read-only in the PDAs, and information cannot be added, erased or modified.

The procedure consists in daily synchronization of the PDAs with the central PC. The PDA database follows the same rules as the rest of the databases; it is backed up regularly and password-protected. The original database is in a PC in the Informatics Office and there is only one copy on the server, which can only be accessed by personnel with access privilege. The design of the tables is relational and the study participant’s ID code is the variable that links with the rest of the databases. Access to the forms in the PDAs are password-protected, as is access to the PDAs themselves.

**II. Databases in the Virology Laboratory**

**A. Data Entry Area**

**General PDVI Database PDVI (General Cohort Data)**

Tables:

Registry of specimen entry

Dengue epidemiological forms

Catalogs of linked data

This database has an interface developed in Visual Basic 6 (Microsoft) that permits validation of which user is using the databases, since each user has a unique password that defines his/her access privileges to the database. This allows registration of which user created the registry and the last user to modify it, along with the date of modification.

**Sample Reception and Separation**

Sample handling starts in the Clinical Laboratory in the Health Center. Information about the sample (e.g., code, date of samples collction, date of symptom onset, category, etc.) is entered into a database from which a barcode label is automatically printed that contains key information.

In the Virology Laboratory, the sample sent from the Clinical Laboratory is received using a Sample Reception database in which information about the date and time of arrival, the temperature of the thermos, the volume of the sample, etc. is recorded. The sample reception is performed using a barcode scanner that reads the information that comes from the Clinical Laboratory and enters it in the registry. This database processes the kind of sample (acute versus convalescent) and automatically prints barcode labels for the tubes. These labels contain a 2-dimensional Data Matrix barcode that contains information about the code of the sample aliquots. In the Sample Separation database, information is stored about the specimen aliquots (e.g., volume, freezer box location, etc.).

**B. Serology Area**

**ELISA database**

Tables:

IgM ELISA results

Inhibition ELISA results

IgG ELISA results

Results of the Annual Sample

Serological results of other projects

Reagent control

**ELISADATA database**

Table of results for approval for each of the techniques.

**Reagent database**

Tables of the reagents used in the Virology Laboratory for ELISA assays.

The ELISA system developed in Visual Basic 6.0 consists of the automatization of the ELISA techniques in the Virology Laboratory. Each user is validated using passwords, and the system permits the use of multiple readers.

In the ELISA system, the technician uses a 2D barcode scanner to generate the electronic work protocol, which reflects the standard work protocol for each of the techniques (including controls, test samples, etc.). This work protocol is printed and stored in an arhive. When the ELISA plate is ready to be read by the ELISA reader, the system (prompted by the technician) sends a signal to the ELISA reader and captures the data in a text file via serial port. This is then formatted to fit the layout of the work protocol (which reflects the ELISA plate), and the results of the assay are presented on the screen with the option to save the data in the system database.

When the technician clicks the button to save the data, the system verifies the control values according to the pre-established criteria. If everything is correct, the system validates the controls and the reagents used in the assay to store the codes along with the results. Then the results sheet is printed, in which all of the information related to the assay is presented (based on the format of the work protocol, but with the results in addition to the sample codes, with the reagent lot information along the bottom). All this information is stored in the database.

**C. Molecular Biology and Cell Culture Areas**

Similar automated work protocols and corresponding databases exist for RT-PCR, viral isolation, and Plaque Reduction Neutralization Test (PRNT).

**D. Handling of the samples from the Annual Sample Collection**

During the annual sample collection, there are two strategies, one in the Health Center auditorium and the other in the field. Study participants who come to the Health Center auditoium first pass through a station for identificaion and registration, where the child’s fingerprint is used to retrieve his/her data, which is presented on the computer screen to verify his/her identity. At this time, the general information of each child is confirmed, and it is verified whether the child is suspected of having dengue at that moment. If not, the computer automatically prints barcode labels for the sample collection tubes (CBC and serology). With these tubes, the participant passes to the nurses’ station when the barcode label is scanned to upload the information to the PDA.

When the sample is collected, the nurse registers pertinent information in the PDA (e.g., volume of sample, code of the person collecting the sample, as well as additional survey information). Periodically, the supervisor receives the samples from each nurses’ station and transfers the accompanying information from their PDAs to the supervisor’s PDA.

In the field strategy, study personnel use GPS, maps and PDAs to locate the participant in his/her home. These visits are directed by GIS and are updated daily such that the field teams visit a pre-selected group of study children; thus, they know which houses they will visit and have the pre-printed barcodes for the sample collection tubes and pre-loaded surveys in their PDAs. When the participant is located, the appropriate barcode labels are placed on the tubes and the sample is collected following established protocols. The same procedure of information registry is followed as in the Health Center auditorium, and the supervisor periodically (every 2-3 hours) receives the samples and the data in the PDAs from the field teams. The supervisor also collects information about the conditions of the samples (e.g., thermos temperature) in both the field and the Health Center auditorium.

The supervisor then brings the samples to the Clincial Laboratory in the Health Center. The samples are received and information uploaded into the database using the barcode label. Information about sample volume and condition is also registered. The CBC sample is then processed using the automated CBC machine.

Verification of data quality is performed by comparison. The information in the PDAs of each nurse team is compared to the reception database of the supervisor, and discrepancies are searched for. The supervisor’s database is compared to the database in the Clinical Laboratory to ensure there are no differences. The CBC reception database is also compared with the Results database to make sure all tubes have been processed.

When the verification process has been completed, the serology samples are sent to the Virology Laboratory. An automatic report is printed listing the samples being sent, which is physically checked against the number of tubes in the thermos. In addition, the database with the information of the samples being delivered is sent to the Virology Laboratory by email. Information about the temperature of the thermos and the time the samples leave the Clinical Laboratory is also registered. At this time, the Clinical Laboratory prints all the CBC results to be returned to the parents of the study participants the following day.

When the samples arrive at the Virology Laboratory, the time and the termperature of the thermos is registered, as well as that of the refrigerator where the samples are stored temporarily. The Virology Laboratory receives the samples in the Reception database using the barcode labels, and this database is compared with what was sent from the Health Center by email. If there are no discrepancies, the labels with 2D barcodes are printed for the separation process, which is carried out using PDAs with embedded barcode scanners. The barcode is used to identify the sample, and the PDA is used to register information about volume and condition of the aliquots.

The storage process is carried out with a database that, using the barcode label, automatically controls what is being stored in the corresponding box. A schematic of the storage box appears on the screen, and as the label on each tube is scanned as it is placed into a slot, that code appears on the diagram of the box on the screen.

For the processing of the annual samples, work procotols are generated automatically according to which the samples are prepared for ELISA. The system automatically processes the information from the ELISA reader and saves the results in the database immediately.

**Communication between sites**

The databases are located in different sites (Health Center and Virology Laboratory), approximately 10 km apart. The results that are shared are the paricipants’ medical consults (sent via email from the Health Center to the Virology Laboratory) and the laboratory results of the dengue tests (sent via email from the Virology Laboratory to the Health Center). To do this, the system constructs tables in a new database, compresses them, and sends them by email; on the other side, the inverse procedure takes place, and the existing database is updated. The databases in each site are updated once a week.
