# Supplementary material for: Integration of Information Technologies in Clinical Studies in Nicaragua
Source: PLoS Med. 2007 Oct 23;4(10):e291. doi: 10.1371/journal.pmed.0040291 (PMC2039765; doi:10.1371/journal.pmed.0040291)
Supplement: Supplementary file 2 [file pmed.0040291.st001.doc]

Table S1. Detailed information about hardware and software including prices

| **Technology** | **Item** | **Manufacturer** | **Price*** |
| --- | --- | --- | --- |
| **PDA** | **Hardware** |  |  |
|  | SPT1550 with integrated barcode scanner | Symbol, Oakland, CA | $415 |
|  | Palm Tungsten | Palm, Sunnyvale, CA | $240 |
|  | Plug-N-Scan Barcode Kit | Portable Technology Solutions, Calverton, NY | $230 |
|  | SPT-1500 Series Recharge Cradle US P/S | Symbol | $135 |
|  | AAA rechargeable batteries and charger, 4-pk | Office Depot | $28 |
|  | **Software** |  |  |
|  | Palm OS v 4.1 or higher | Palm, Sunnyvale, CA | Free |
|  | HanDBase Professional Edition database manager | DDH Software, Wellington, FL | $40 |
|  | Pendragon Forms 5.0 | Pendragon Software Corporation, Libertyville, IL | $229 |
|  | - Additional Handheld User Licenses/ea | Pendragon Software Corporation | $60 |
| **Fingerprint** | **Hardware** |  |  |
|  | U.are.U 4000 scanner | Digital Personna, Redwood City, CA | $95 |
|  | **Software** |  |  |
|  | VeriFinger 4.2 SDK Standard (plus scanner) | Neurotechnologija, Vilnius, Lithuania http://www.neurotechnologija. com/ verifinger.html | $500 |
|  | - 5 software licenses and 5 scanners | Neurotechnologija | $1,020 |
| **Barcodes** | **Scanners** |  |  |
|  | MS9520 Voyager: 1D Applications | Metrologic, West Deptford, NJ | $180 |
|  | Opticon LG2: 2D Applications | Opticon USA, Bellevue, WA | $700 |
|  | **Printers and accessories** | Zebra, Vernon Hills, IL |  |
|  | Thermal transfer printer: Zebra TLP 2844 |  | $420 |
|  | TR 220 portable printer adaptor |  | $500 |
|  | 5095 universal resin ribbons, 2.25” x 244’ |  | $6 |
|  | **Labels** | Zebra, Vernon Hills, IL |  |
|  | Polypro 1000, 1.5” x 0.5”, 3780/roll |  | $23 |
|  | Cryocool 3000, 1.5” x 0.75”, 3000/roll |  | $50 |
|  | **Software** |  |  |
|  | Print Studio Professional 2.0 | Jolly Technologies, San Carlos, CA | $400 |
|  | Tracer Plus Professional | Tracerplus Co, UK | $160 |
|  | - Additional License | Tracerplus Co | $90 |
| **GIS** | GPS: GARMIN 12, GARMIN 60, Garmin e-Trex | Garmin, Olathe, KS | $200-300 |
|  | **Software** |  |  |
|  | MapSource from Garmin |  | Free |
|  | ArcView GIS 9.0 | ESRI, Redlands,CA | $1,500 |
| **Other** | Computers | Dell | ~ $1,000 |
|  | Visual Studio |  | $1,200 |
|  | Microsoft Office |  | ~ $500 |

Prices were actual in 2006; however, they change by vendor and year
